# Sepsis Bundle Adherence Is Associated with Improved Survival in Severe Sepsis or Septic Shock

**DOI:** 10.5811/westjem.2018.7.37651

**Published:** 2018-08-13

**Authors:** Peter K. Milano, Shoma A. Desai, Erick A. Eiting, Erik F. Hofmann, Chun N. Lam, Michael Menchine

**Affiliations:** LAC+USC Medical Center, Keck School of Medicine at University of Southern California, Department of Emergency Medicine, Los Angeles, California

## Abstract

**Introduction:**

There have been conflicting data regarding the relationship between sepsis-bundle adherence and mortality. Moreover, little is known about how this relationship may be moderated by the anatomic source of infection or the location of sepsis declaration.

**Methods:**

This was a multi-center, retrospective, observational study of adult patients with a hospital discharge diagnosis of severe sepsis or septic shock. The study included patients who presented to one of three Los Angeles County Department of Health Services (DHS) full-service hospitals January 2012 to December 2014. The primary outcome of interest was the association between sepsis-bundle adherence and in-hospital mortality. Secondary outcome measures included in-hospital mortality by source of infection, and the location of sepsis declaration.

**Results:**

Among the 4,582 patients identified with sepsis, overall mortality was lower among those who received bundle-adherent care compared to those who did not (17.9% vs. 20.4%; p=0.035). Seventy-five percent (n=3,459) of patients first met sepsis criteria in the ED, 9.6% (n=444) in the intensive care unit (ICU) and 14.8% (n=678) on the ward. Bundle adherence was associated with lower mortality for those declaring in the ICU (23.0% adherent [95% confidence interval{CI} {16.8–30.5}] vs. 31.4% non-adherent [95% CI {26.4–37.0}]; p=0.063), but not for those declaring in the ED (17.2% adherent [95% CI {15.8–18.7}] vs. 15.1% non-adherent [95% CI {13.0–17.5}]; p=0.133) or on the ward (24.8% adherent [95% CI {18.6–32.4}] vs. 24.4% non-adherent [95% CI {20.9–28.3}]; p=0.908). Pneumonia was the most common source of sepsis (32.6%), and patients with pneumonia had the highest mortality of all other subsets receiving bundle non-adherent care (28.9%; 95% CI [25.3–32.9]). Although overall mortality was lower among those who received bundle-adherent care compared to those who did not, when divided into subgroups by suspected source of infection, a statistically significant mortality benefit to bundle-adherent sepsis care was only seen in patients with pneumonia.

**Conclusion:**

In a large public healthcare system, adherence with severe sepsis/septic shock management bundles was found to be associated with improved survival. Bundle adherence seems to be most beneficial for patients with pneumonia. The overall improved survival in patients who received bundle-adherent care was driven by patients declaring in the ICU. Adherence was not associated with lower mortality in the large subset of patients who declared in the ED, nor in the smaller subset of patients who declared in the ward.

## INTRODUCTION

The Surviving Sepsis Campaign (SSC) has established internationally endorsed guidelines for the management of patients with severe sepsis or septic shock (referred to as “sepsis” throughout this article).^1^ These guidelines are distilled into bundles, which combine various components of sepsis care such as fluid resuscitation, timely and appropriate antibiotics, blood cultures, and the use of serum lactate levels. These components have evolved into core measures put forth by the Centers for Medicare & Medicaid Services (CMS) in October 2015. As hospital compensation from CMS is partially dependent on quality measure performance, hospital administrative efforts and resources have been directed toward improved compliance and accurate reporting. Due to the complexity of requirements and data verification procedures, it is estimated that hundreds of thousands to millions of dollars per year per hospital are spent on meeting and reporting these measures.^2^

The clinical benefit of adherence should be clearly demonstrated to justify this costly effort, but there are reasons for skepticism. In fact, some CMS quality metrics related to acute infections have had undesired negative effects. For example, the quality measure “blood cultures performed in the ED prior to initial antibiotics received in the hospital” for pneumonia^3^–^5^ has been shown to be costly, results in high false-positive blood culture rates, and rarely results in antibiotic changes while simultaneously prolonging hospital length of stay.^4^,^6^

To date, experiences with the sepsis bundles have been mixed. Some studies have demonstrated an improvement in overall mortality with sepsis-bundle adherence,^1^,^7^,^8^ but some of the most prominent recent studies examining sepsis treatment, including the ProCESS, ProMISe and ARISE trials, failed to show a similar benefit.^9^–^13^ These contradictory findings may be due to smaller sample sizes, heterogenous effects of bundle adherence based on the source of infection (e.g., bundle adherence may matter more for pneumonia than urinary tract infection [UTI]), or variability in the site of sepsis declaration in the hospital (ED vs. intensive care unit [ICU]).

Using best practices from the SSC, the the Los Angeles County Department of Health Services (DHS) implemented an initiative to improve sepsis management through the use of bundles at its public hospitals. The strategy developed by DHS to improve sepsis care included implementation of a resuscitation bundle, measuring and assuring adherence with the bundle, and tracking mortality for patients with sepsis. Using data archived throughout this process, the current study sought to achieve the following: 1) characterize the association between bundle adherence and mortality for patients with sepsis; 2) examine whether the location of declaration in the hospital (ED vs. ward vs. ICU) impacts the relationship between bundle adherence and mortality; and 3) explore how the source of infection influences the relationship between bundle adherence and mortality.

**Population Health Research Capsule**

What do we already know about this issue?

Resources are being expended by hospitals on sepsis-bundle quality measures. It is unclear which patients with sepsis benefit from adherence to these bundles.

What was the research question?

Is sepsis-bundle adherence associated with improved mortality? Does location of declaration or source of infection matter?

What was the major finding of the study?

Sepsis-bundle adherence was associated with lower mortality in intensive care unit declarations, but not in cases declaring in the emergency department or ward.

How does this improve population health?

Focusing resource-intensive treatments on patients who benefit improves the value of care. This study explores which hospitalized patients benefited from sepsis-bundle adherence.

## METHODS

This was a multi-center, retrospective, observational study of adult patients (≥ 18 years old) with a hospital discharge diagnosis of either severe sepsis or septic shock (ICD-9). The study included patients who presented to one of three Los Angeles County DHS full-service inpatient hospitals following implementation of a sepsis improvement initiative. Beginning in 2011, the sepsis program was implemented in phases across these sites. Excluding this staggered roll out, the study period encompasses January 2012 through December 2014. This study was approved by the DHS institutional review board.

We included in the dataset patients meeting severe sepsis or septic shock clinical criteria (Table 1) within the ED or inpatient setting. The inclusion criteria for severe sepsis was suspected or confirmed infection, two or more systemic inflammatory response syndrome (SIRS) criteria, and evidence of acute organ dysfunction. SIRS criteria included the following: body temperature > 38 ºC or < 36 ºC; heart rate > 90 beats per minute; respiratory rate > 20 respirations per minute or partial pressure of carbon dioxide in arterial blood (PaCO_2_) < 32 mmHg, and white blood cell count > 12,000 per mm^3^ or < 4,000 per mm^3^ or a bandemia of > 10%. Organ dysfunction was defined as a new-onset ventilator requirement, vasopressor requirement, new-onset creatinine elevation > 2 mg/dL, new-onset INR > 1.5 in the absence of warfarin, FiO_2_ > 30%, or new-onset thrombocytopenia of < 100,000 per μL. Septic shock was defined as severe sepsis plus lactate ≥ 4 mmol/L and/or systolic blood pressure < 90 mmHg or mean arterial pressure < 65 mmHg after 20 mL/kg of crystalloid fluid. Patients receiving comfort care were excluded.

Bundle adherence metrics were adapted from the SSC bundles from 2012.^1^ For the purposes of this research project, bundle adherence was defined as the following: 1) lactate levels drawn within four hours pre-declaration or six hours post-declaration; 2) blood cultures prior to antibiotic administration; 3) a minimum of 20mL/kg of crystalloid fluids administered within six hours pre-declaration or six hours post-declaration (Patients with documented evidence of fluid overload were exempt from the intravenous fluid administration requirement. Fluid overload was defined as pulmonary edema on chest radiograph, an elevated B-natriuretic peptide level, or documentation of a plethoric inferior vena cava on bedside ultrasound.); 4) antibiotics administered within three hours of declaration in the ED setting or within one hour of declaration in the inpatient setting. Bundle adherence for patients in septic shock included the above components plus the administration of vasopressors.

Trained, utilization-review nurses recorded location of sepsis declaration (ED vs. ICU vs. ward) and timestamps associated with administration of antibiotics, completion of target fluid administration, and measurement of serum lactate levels. They determined the source of infection by reviewing the admission and discharge diagnoses and reviewing laboratory and radiographic data. These event data were used by the researchers to assess for adherence to the bundle.

The primary outcome analyzed was in-hospital mortality. Secondary outcome measures included in-hospital mortality by source of infection and location of declaration. Descriptive statistics were generated for all variables with appropriate confidence intervals. We used chi-square and Mann-Whitney U-tests of statistical significance for categorical and continuous variables, as appropriate.

## RESULTS

Demographics of the study population are listed in Table 2. The mean age was 54.8 years and the median age was 55.5. Further, 75.5% (n=3,459) declared in the ED, 9.6% (n=444) declared in the ICU, and 14.8% (n=678) declared on the ward. Pneumonia was the most common source of infection (32.6%; n=1,494) followed by UTI (20.3%; n=929). Overall in-hospital mortality was 18.9% (n=867) and overall bundle adherence was 60.1% (n=2,755).

Overall, sepsis-bundle adherence was associated with improved mortality. Mortality among patients with bundle adherence care was 17.9% (95% CI [16.5–19.4]), whereas it was 20.4% (95% CI [18.6–22.3]) for those who did not receive bundle-adherent care. The relative increase in mortality rate for bundle non-adherence as compared with bundle adherence was 14.0% as is shown in Table 3. In general, regardless of the anatomic origin of sepsis, mortality was improved for patients who received bundle adherent care. Pneumonia had a relative increase in mortality rate for non-adherence of 36.3% (p<0.001), followed by multiple sources (33.2%; p=0.165), intra-abdominal/gynecologic (16.6%; p=0.347), and UTI (6.6%; p=0.804).

The mortality improvement for bundle-adherent care was not consistent across all sites of sepsis declaration (as shown in Table 4). Bundle adherence was associated with a trend toward improved mortality for patients whose sepsis declared in the ICU (23.0% adherent [95% CI {16.8–30.5}] vs. 31.4% non-adherent [95% CI {26.4–37.0}]; p=0.063), but was similar in ED declarations (17.2% adherent [95% CI {15.8–18.7}] vs. 15.1% non-adherent [95% CI {13.0–17.5}]; p=0.133) and in ward declarations (24.8% adherent [95% CI {18.6–32.4}] vs 24.4% non-adherent [95% CI {20.9–28.3}]; p=0.908).

**Figure 1:**
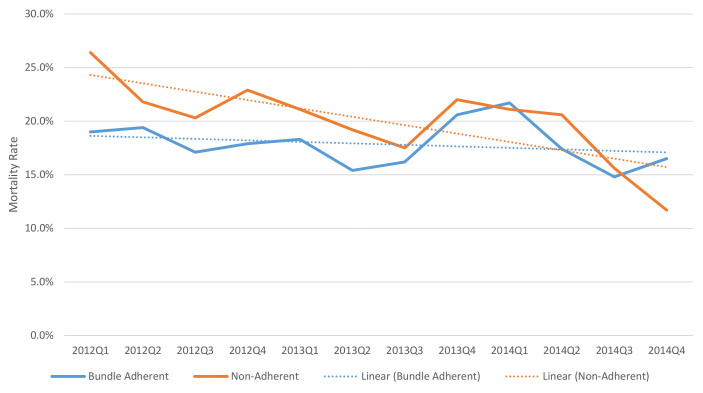
Mortality rates by bundle adherence, overall, 2012–2014.

Figure 1 depicts the relative rate of mortality for bundle adherent and bundle non-adherent patients per quarter from January 2012 to December 2014. The mortality over time of patients receiving bundle adherent care is generally lower than the mortality of those receiving non-adherent care.

**Figure 2:**
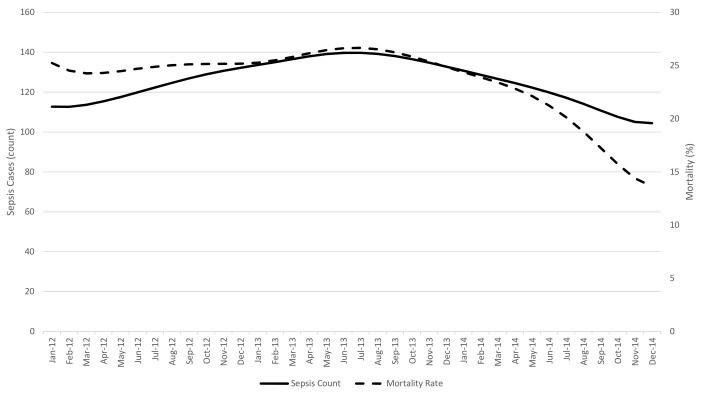
Sepsis cases (count) vs. mortality rate over time, LOWESS* smoothing trend lines. *LOWESS*, locally weighted scatterplot smoothing.

A locally-weighted scatterplot smoothing comparing month-to-month sepsis cases vs. mortality rate is depicted in Figure 2. Although there was some fluctuation in the number of sepsis cases from month-to-month, overall the number of sepsis cases remained relatively stable from January 2012 through December 2014 (ranging from 104 to 140) while the mortality rate decreased. We saw an initial trend toward more sepsis cases from January 2012 through July 2013 with a high of 140 cases for the month of July 2013. The mortality rate remained relatively stable from January 2012 through September 2013 with an average rate of 25.3%. The rate then steadily decreased from 26.2% in September 2013 to 13.6% by December 2014.

## DISCUSSION

CMS implemented the Severe Sepsis and Septic Shock Management Bundle in 2015, thus establishing a significant financial incentive for adherence, despite conflicting scientific evidence on its clinical impact. In this large study of a major urban healthcare system, we found that bundle-adherent care was, in fact, associated with lower mortality overall. Interestingly, the effect of bundle adherence varied markedly depending on the location of declaration. In the ICU, a 7% absolute decrease in mortality was associated with bundle adherence. Conversely in the ward and ED, bundle adherence was not associated with any improvement in mortality. This finding merits careful exploration as the great majority (75.5%) of sepsis patients were diagnosed in the ED.

It is unclear why bundle adherence did not have an association with improved mortality for ED patients but did for ICU patients (though not statistically significant). One possibility is that patients presenting to the ED with severe sepsis or septic shock had been suffering from the condition for many hours to days but could only “declare” once they arrived for medical attention. As a result, the marginal advantage of “timely care” per bundle requirements as compared with the timeline of disease evolution outside of the hospital was lost. For patients declaring in the ICU, it may be that they truly developed sepsis contemporaneous with the declaration of sepsis and that, therefore, early intervention was possible. Another factor may be that patients declaring in the ICU had higher illness severity, making treatment effects more easily observed. A final possibility is that “non-adherent” care in the ED may have been *almost* adherent care, perhaps only missing quality goals by a few minutes or few milliliters. In such circumstances, any mortality differences between adherent and non-adherent subjects would have been muted.

We found only two other studies that specifically examined the relationship between sepsis-bundle adherence and mortality in ED patients that yielded conflicting results. One small study (N=117) from Singapore found no statistically significant relationship between bundle compliance and mortality for ED patients,^14^ while another study (N=330) observed a very large difference in mortality between bundle adherent and non-adherent ED patients with sepsis.^15^ It should be noted that the current study is roughly 10 times larger than these previous two combined. Determining whether bundle adherence in the ED leads to improved patient outcomes is a matter of urgent importance. Between 50–75% of all sepsis cases declare in the ED, and consequently, a major emphasis of clinical and administrative work is geared toward ensuring bundle adherence in this area of care. These are efforts that could perhaps be better focused on the ICU setting for greater clinical impact. Future research should attempt to replicate our findings across a broad array of ED settings.

Another goal of this investigation was to explore whether the anatomic source of infection influenced the effect of sepsis-bundle adherence. We found that pneumonia was the most common source of sepsis (32.6%), and bundle-adherent care was associated with lower mortality in such cases (28.9% non-adherent vs. 21.2% adherent), a relative increase in mortality rate for non-adherence of 36.3%. This finding aligns with other investigations demonstrating an association between timely antibiotic treatment and improved mortality in pneumonia cases.^16^ Interestingly, we observed a non-significant trend toward a reduction in relative mortality for other anatomic sources of infection (e.g. UTI, intra-abdominal/gynecologic, and multiple sources) with the exception of sepsis due to bone/soft tissue/wound infections and unknown sources. This may reflect the small number of patients within these subgroups rather than a true difference in the impact of bundle adherence across different sources of infection. Ultimately, our findings support the practice of attempting to provide bundle-adherent care for all patients with sepsis regardless of the suspected anatomic site of infection.

One notable observation in our study was the relative lack of increased sepsis cases over time. A criticism of existing sepsis literature is that increasing sensitivity of diagnosis over a study period may artificially lower mortality calculations. With an increasing awareness of sepsis, there should be an increase in the diagnosis of marginal or early sepsis cases. If such marginal cases (presumably of lower acuity) were incorporated into the data pool while bundle adherence was generally improving over time, one would expect to observe an association between compliance and mortality that would be confounded by severity.^17^,^18^ In our study, however, a decreased absolute patient mortality was noted while the number of sepsis cases remained relatively stable over time. This supports the observation that improving bundle adherence is associated with decreased mortality and is not simply a result of enhanced documentation.

## LIMITATIONS

This study is subject to limitations inherent in a retrospective study design. Though our abstractors were blinded to study objectives, it is possible that they were influenced by administrative pressure to meet bundle-adherence goals. To minimize this limitation, we used timestamps at the patient level and recalculated intervals and bundle adherence. Even if these biases influenced the documentation of events, they likely would not have impacted mortality rates substantially. It should also be noted that there are intrinsic differences between public and community hospitals. Decreased access to preventative care, prolonged ED wait times and increased ED boarding times is an unfortunate but constant reality in today’s public hospitals.^19^,^20^ It is possible that a greater percentage of patients declare in the ED when their disease course is in a more advanced stage due to lack of insurance.

The CMS SEP-1 Core Measure requirements at the time of the publication of this study^21^ are different from the severe sepsis and septic shock criteria used during this study. Significant differences include the following: increasing the fluid administration requirement from 20mL/kg crystalloid to 30mL/kg; the current lack of an exemption from fluid boluses in the context of clinical evidence of fluid overload; and the inclusion of lactate >2 as a criteria for acute organ dysfunction (and therefore severe sepsis). The data presented in this study may not reflect the effects of the SEP-1 interventions.

Another potential limitation is that the source of infection was established through chart review by the abstractors. Patients with confounding laboratory, radiographic or diagnosis codes may have been mis-categorized into source of infection. Finally, we did not have the clinical detail to calculate severity indices (e.g. APACHE scores). Without these clinical data, it was impossible to discern whether there were differences in severity of illness in the bundle adherence and bundle non-adherence groups. As such, more severely ill patients could have been managed differently than those who were less severely ill. This possibility limits the conclusions of the study. Further prospective analysis using severity indices is warranted.

## CONCLUSION

In a large public healthcare system, adherence with severe sepsis/septic shock management bundles was associated with an overall improvement in survival. This was generally true regardless of the anatomic site of infection. Interestingly, the mortality benefit of bundle-adherent care was concentrated in ICU patients; and we did not observe any benefit to bundle-adherent care for patients with sepsis in the ED or in those who declared on the hospital ward. Further study to determine the importance of sepsis-bundle adherence is especially needed in the ED setting, given that the great majority of sepsis cases are declared there.

## Figures and Tables

**Table 1:** Definitions of severe sepsis, septic shock and bundle adherence.

Severe sepsis	Septic shock	Bundle adherence
Suspected or confirmed infection	Severe sepsis plus lactate ≥ 4 mmol/L	Lactate levels drawn within 4 hours pre-declaration or 6 hours post-declaration
Two or more SIRS criteria^A^	AND/OR systolic blood pressure < 90 mmHg or mean arterial pressure < 65 mmHg after 20 mL/kg of crystalloid fluid	AND blood cultures prior to antibiotic administration
Evidence of acute organ dysfunction^B^		AND a minimum of 20mL/kg of crystalloid fluids administered within 6 hours pre-declaration or 6 hours post-declaration^C^
		AND antibiotics administered within 3 hours of declaration in the ED setting, or within 1 hour of declaration in the inpatient setting
		AND administration of vasopressors, if in septic shock

*SIRS*, systemic inflammatory response syndrome; *mmol/L*, millimoles per liter; *mmHg*, millimeters of mercury; *mL/kg*, milliliters per kilogram; *ED*, emergency department; *mm^3^*, millimeters cubed.

A SIRS criteria included temperature > 38 °C or < 36 ºC, heart rate > 90 beats per minute, respiratory rate > 20 respirations per minute or partial pressure of carbon dioxide in arterial blood (PaCO_2_) < 32 mmHg, and white blood cell count > 12,000 per mm3 or < 4,000 per mm^3^ or a bandemia of > 10%.

B Acute organ dysfunction was defined as new-onset ventilator requirement, vasopressor requirement, new-onset creatinine elevation > 2 mg/dL, new-onset INR > 1.5 in the absence of warfarin, FiO_2_ > 30%, new-onset thrombocytopenia of < 100,000 per μL.

C Patients with documented evidence of fluid overload were exempt from the intravenous fluid administration requirement. Fluid overload was defined as pulmonary edema on chest radiograph, an elevated B-natriuretic peptide level, or documentation of a plethoric inferior vena cava on bedside ultrasound.

**Table 2:** Demographic characteristics of patients (N=4,582).

Patient demographics	N	%
Gender		
Male	2451	53.8
Female	2106	46.2
Race		
Asian	416	9.1
African American	593	13
White	3017	66.2
Other	389	8.5
Unknown	143	3.1
Ethnicity		
Hispanic or Latino	2783	61
Not Hispanic or Latino	1623	35.6
Unknown	153	3.4
Language		
English	2259	49.6
Spanish	2042	44.8
Other	256	5.6
Marital status		
Married	1280	27.9
Single	2332	50.9
Widowed/divorced/separated	720	15.7
Unknown	206	4.5
Location of declaration		
Emergency department	3459	75.50%
Intensive care unit	444	9.60%
Ward	678	14.80%
Facility		
LAC+USC	1965	42.90%
HUCLA	1447	31.60%
OVMC	1170	25.50%
Source of infection		
Pneumonia	1494	32.60%
Urinary tract infection	929	20.30%
Abdominal/gynecologic	606	13.20%
Bone/soft tissue/wound	481	10.50%
Multiple sources	317	6.90%
Unknown source	755	16.50%
Bundle adherent care	2755	60.10%
Mortality	867	18.90%

*LAC+USC*, Los Angeles County + USC Medical Center; *HUCLA*, Harbor-UCLA Medical Center; *OVMC*, Olive View Medical Center.

**Table 3:** Mortality rate by bundle adherent vs. non-adherent, per infection source.

		Bundle adherent		Bundle non-adherent			
					
Source of infection	Total cases (n, %)	% Mortality	95% CI	% Mortality	95% CI	Relative increase in mortality rate for non-adherence	P value
Overall	4582	17.9%	16.5, 19.4	20.4%	18.6, 22.3	14.0%	0.035
Pneumonia	1494 (32.6%)	21.2%	18.7, 23.9	28.9%	25.3, 32.9	36.3%	<0.001
Urinary tract infection	929 (20.3%)	6.1%	4.3, 8.4	6.5%	4.4, 9.4	6.6%	0.804
Abdominal/gynecologic	606 (13.2%)	18.7%	14.8, 23.3	21.8%	17.3, 27.0	16.6%	0.347
Bone/soft tissue/wound	481 (10.5%)	13.0%	9.5, 17.6	11.7%	7.9, 16.9	−10.0%	0.661
Multiple sources	317 (6.9%)	20.2%	15.0, 26.7	26.9%	20.0, 35.1	33.2%	0.165
Unknown source	755 (16.5%)	26.5%	22.7, 30.7	25.1%	20.4, 30.5	−5.3%	0.672

*CI*, confidence interval.

**Table 4:** Mortality rate by site and bundle adherence.

		Died during hospitalization		Survived hospitalization		
				
Location of sepsis declaration	Bundle adherence	n, %	95% CI	n, %	95% CI	P value
Emergency department						
	(+) Bundle adherence	422 (17.2%)	15.8, 18.7	2031 (82.8%)	81.3, 84.2	0.133
	(−) Bundle adherence	152 (15.1%)	13.0, 17.5	854 (84.9%)	82.5, 87.0	
Ward						
	(+) Bundle Adherence	38 (24.8%)	18.6, 32.4	115 (75.2%)	67.6, 81.4	0.908
	(−) Bundle adherence	128 (24.4%)	20.9, 28.3	397 (75.6%)	71.7, 79.1	
Intensive care unit						
	(+) Bundle adherence	34 (23.0%)	16.8, 30.5	114 (77.0%)	69.5, 83.2	0.063
	(−) Bundle adherence	93 (31.4%)	26.4, 37.0	203 (68.6%)	63.0, 73.6	
Overall						
	(+) Bundle adherence	494 (17.9%)	16.5, 19.4	2,260 (82.1%)	80.6, 83.5	0.036
	(−) Bundle adherence	373 (20.4%)	18.6, 22.3	1,454 (79.6%)	77.7, 81.4	

*CI*, confidence interval.
